# *Candida blankii*: The Difficult Capture of a Fungus With Pathogenic Potential

**DOI:** 10.1155/crdi/5543654

**Published:** 2025-10-23

**Authors:** Ethan Smillie, Arjun Sharma, Johan Delport, Ana Cabrera, Mohammedreza Rahimi Shahmirzadi, Fatimah AlMutawa

**Affiliations:** ^1^Faculty of Science Department, Schulich School of Medicine & Dentistry, Western University, London, Ontario, Canada; ^2^Department of Medicine, Division of Infectious Diseases, Western University, London, Ontario, Canada; ^3^Department of Pathology and Laboratory Medicine, Division of Medical Microbiology, Western University, London, Ontario, Canada; ^4^Department of Microbiology and Immunology, Western University, London, Ontario, Canada

**Keywords:** *Candida blankii*, fungemia, pathogen

## Abstract

*Candida blankii* has recently emerged as a pathogen of clinical significance, particularly in cases of candidemia. Here, we present two cases involving adult patients with complex medical histories. In one case, *C. blankii* was considered clinically significant, while in the other, it was regarded as a colonizer. The first case involves an 85-year-old male with multiple comorbidities, including chronic obstructive pulmonary disease and heart failure, who presented with a pleural effusion. Blood cultures revealed yeast which could not be identified by matrix-assisted laser desorption/ionization time-of-flight mass spectrometry (MALDI-TOF MS), which was later identified as *C. blankii* after being sent to the provincial reference laboratory. The isolate showed high minimum inhibitory concentrations (MICs) to azoles. The second case involves a 60-year-old male with cirrhosis and multifocal pneumonia. *C. blankii* was isolated from bronchoalveolar lavage samples, though it was ultimately considered a colonizer rather than a pathogen in this instance. Initial identification via MALDI-TOF MS was inconclusive, necessitating further molecular sequencing. The pathogen exhibited high MICs to azoles and lower MICs to echinocandins and polyenes. Both cases highlight the challenges in identifying *C. blankii* using conventional laboratory methods. Given the increasing reports of *C. blankii* as a pathogen, particularly in immunocompromised patients, our findings emphasize the need for heightened awareness and improved diagnostic techniques. Accurate and timely identification is crucial for appropriate therapeutic management, given the organism's unique susceptibility profile. Further research is necessary to understand the epidemiology, pathogenesis, and optimal treatment strategies for *C. blankii* infections.

## 1. Introduction

Over the past decade, there have been increasing reports of infections due to the yeast *Candida blankii* (*C. blankii*). The yeast was originally isolated from infected Canadian mink in 1968 by scientists Buckley and Uden [1]. It appears first in the literature to be associated with an invasive human infection as part of a national survey of candidemia in persons in Norway in the 1990s [2]. In recent years, *C. blankii* has been isolated as a bloodstream pathogen in neonates and adults and in the respiratory tracts of patients with underlying pulmonary pathologies. All cases emphasized difficulty with methods employed to initially identify the organism, which in turn demonstrated a reduced susceptibility to azole-based agents compared to other antifungal therapies such as echinocandins and polyenes. As an emerging fungal pathogen of clinical significance, here we describe two cases in adults—the first reported human cases in Canada.

## 2. Case 1

**Figure 1:**
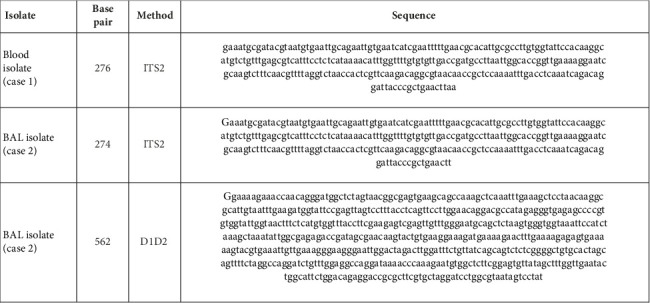
Nucleotide sequences.

An 85-year-old man presented to our tertiary care hospital with shortness of breath and radiographic evidence of a loculated right pleural effusion. His medical history was significant for chronic obstructive pulmonary disease, heart failure, peripheral arterial disease, recurrent lower extremity cellulitis, and a necrotizing fasciitis that previously required a right above knee amputation. He was afebrile, and his physical exam demonstrated decreased air entry over the right lower lung field, and pitting edema to the left tibial tuberosity and in the right residual limb. While no respiratory samples were obtained, blood cultures were positive for yeast in one of two sets. The wet mount examination revealed small budding yeasts. These yeasts grew on blood agar and were subcultured on Sabouraud agar (white cream colonies with smooth surface) and Candida Select plates. The Dalmau plate technique, which uses corn meal agar, showed no pseudohyphae or chlamydospores, and the urease test was negative. Repeated analyses using MALDI-TOF mass spectrometry (MALDI Biotyper DB (RUO) V12.000) identified multiple *Candida* species, but the results were inconclusive due to low scores and insufficient score differentiation among the species. Patient was started empirically on intravenous high-dose fluconazole (800 mg loading dose IV then 400 mg IV daily) on admission Day 3. An ophthalmologic exam found no evidence of ocular candidiasis, and both transthoracic echocardiogram (TTE) and transesophageal echocardiogram (TEE) did not demonstrate endocarditis. For the concern of an emerging yeast, his therapy was switched to caspofungin on admission Day 7 to complete a 14-day course. Since conventional MALDI-TOF MS could not characterize the organism, the sample was sent to the provincial reference laboratory and *C. blankii* was identified 3 weeks after the initial specimen was collected. The blood isolate underwent internal transcribed spacer (ITS) sequence analysis. The ITS2 region (276 bp) identified *C. blankii* based on alignment with nucleotide databases. BLAST results showed a similarity of 97.83% for *C. blankii* in both the National Center for Biotechnology Information (NCBI) and the Centraalbureau voor Schimmelcultures (CBS) database. Further alignment with NCBI-type strains showed 100% identity to *C. blankii* (Figure 1). Using the methods described by Clinical and Laboratory Standards Institute method CLSI M27-M44S, the isolate demonstrated high minimum inhibitory concentrations (MICs) to azoles (fluconazole 64 μg/mL, itraconazole 0.5 μg/mL, posaconazole 0.25 μg/mL, and voriconazole 1 μg/mL) and low MICs to echinocandins (caspofungin 0.06 μg/mL, micafungin 0.06 μg/mL, and anidulafungin 0.12 μg/mL) and polyenes (amphotericin B 0.5 μg/mL); no clinical interpretations are available for this species.

## 3. Case 2

A 60-year-old man presented with encephalopathy and radiographic evidence of multifocal pneumonia. His medical history was notable for cirrhosis secondary to end-stage metabolic dysfunction-associated steatotic liver disease, and he was listed for a liver transplant. Upon examination, he was afebrile but required BiPAP support. His physical exam revealed an altered mental state, diminished air entry at the bilateral basal lung fields, and anasarca. Empirical therapy was initiated with imipenem and intravenous azithromycin.

On Day 9 of admission, a bronchoscopy was performed, and bronchoalveolar lavage (BAL) specimens were collected. The cultures from these specimens returned positive for *Enterobacter cloacae* complex and yeast, while PCR detected parainfluenza virus. Patient was started empirically on imipenem (1 g every 8 h) to treat the *E. cloacae* complex; however, subsequent testing determined only intermediate susceptibility which prompted a change in therapy. Imipenem was discontinued, and targeted treatment with meropenem (1 g every 8 h) was administered for 14 days to treat the *Enterobacter cloacae* complex, and azithromycin was continued for 10 days to empirically cover atypical organisms (with BAL cultures for *Legionella*, *Mycoplasma*, and *Ureaplasma* later returning negative).

The BAL culture also grew yeast, which appeared as small budding yeast and formed cream-colored colonies on Sabouraud agar. Multiple attempts to identify the yeast via MALDI-TOF MS were unsuccessful. The isolate was subsequently sent to the provincial reference laboratory, where it was identified as *Candida blankii* 16 days after the initial specimen collection. Clinically, the yeast was considered to represent colonizing flora, no antifungal treatment was administered, and blood cultures showed no growth. The BAL isolate was subject to ITS2 sequence analysis (274 bp), with BLAST analysis yielding a 100% match to *C. blankii* (NCBI), and both CBS and UNITE databases demonstrated 97.8% similarity. Further D1D2 sequence analysis (562 bp) showed 99.6% similarity to *C. blankii* in both NCBI and CBS databases using BLAST analysis, with a query cover of 98%. Alignment with type strain sequencing also confirmed 99.6% identity, and the closest nonblankii match was *Candida digboiensis* (Figure 1).

## 4. Discussion

Candidemia remains a significant cause of bloodstream infections in hospitalized patients. In the United States, the Centers for Disease Control and Prevention (CDC) previously estimated that 22,660 cases of candidemia occur annually [3]. CDC has more recently identified a total of 7381 candidemia cases throughout the surveillance period of 2017–2021 with an incidence of 7.4 cases per 100,000 persons [4]. Candida species other than *Candida albicans* (*C. albicans*) account for roughly half of such infections, and the overall mortality from candidemia is upwards of 30% at 30 days [5, 6]. While *Candida albicans* remains the most common species, there is an increasing burden of nonalbicans species, among which *Candida blankii* (also known as *Tardiomyces blankii*) [7] represents an exceedingly rare cause. In a recent multicenter genomic study proposed by Spruijtenburg et al. [7], 26 isolates reported as C. blankii were analyzed, with 19 confirmed as *T. blankii* and 7 reclassified as the novel species *T. depauwii*. Because of the large phylogenetic difference to *Candida* species, the authors proposed reclassification to this new genus.

Our report of two cases of *C. blankii* in adults is thus significant and aligns with, and contributes to, the rare cases that have been previously documented (Table 1). As with our first case, its main pathogenic potential appears to be as a bloodborne pathogen in both neonates and adults, a presentation described by Chowdhary et al. [8], Al-Haqqan et al. [9], Kollu et al. [12], and Mirchin et al. [13]. Conversely, as with our second case, *C. blankii* is postulated to be a colonizer of the respiratory tract where its role may be more in keeping with an opportunistic pathogen. Although clinical findings in the second case are more consistent with colonization rather than active invasive disease, the patient's advanced age and history of liver cirrhosis necessitate vigilance for possible progression. The divergent clinical manifestations between the two cases align with the damage–response framework proposed by Casadevall and Pirofski [15], which suggests that disease pathogenesis results from the dynamic interaction between microbial attributes and host immune status, rather than from the presence of the microorganism alone. In Case 1, advanced age may have impaired host immune competence, predisposing to a more invasive and virulent bloodstream infection. In contrast, Case 2 involved the respiratory tract—a site where *Candida* species more commonly represent colonizers—alongside the detection of parainfluenza virus and *Enterobacter cloacae* complex, which were managed. These concomitant pathogens, combined with an apparently intact host immune response, may have limited *Candida blankii* to a noninvasive, colonizing role in this pulmonary setting. Nobrega de Almeida et al. [11] surmise that a heavy respiratory colonization of *C. blankii* in a 16 year old with cystic fibrosis was the source of her fungemia, after she underwent a bilateral lung transplant and had her antifungal prophylaxis discontinued due to its adverse effects. Moreover, Zaragoza et al. [10] reported a 14 year old with cystic fibrosis with recurrent pulmonary exacerbations and worsening lung function whose condition only improved with targeted treatment of the *C. blankii* that was isolated from their BAL. Nevertheless, pulmonary candidiasis is a rare phenomenon whose virulence is not always readily apparent [16].

Across the literature of infections from *C. blankii*, a consensus on its source within the human flora was not achieved. Of note, *C. blankii* was isolated from the damaged skin of a patient with atopic dermatitis in Russia by Arzumanyan [17]. This could, in part, account for the pathogenesis of candidemia in our first case, whereby a disrupted integument from recurrent bouts of cellulitis and peripheral arterial disease create portals of entry. The ability of *C. blankii* to tolerate high temperature and salinity environments for its growth make humans also acceptable hosts [6]. It may also suggest an emergence tied to climate change and global warming, the theory proposed for the rise of *C. auris* [18], as *C. blankii* has been isolated from the floral nectar of insect-pollinated plants [19].

*C. blankii* is different than *C. albicans* in several ways. First, in inoculated *Galleria mellonella* larvae models with *C. blankii* and *C. albicans*, the former was found to be less virulent as evidenced by a greater percent survival over 7 days [11]. *C. blankii* typically forms yeast-like colonies that are white to cream in color, with a smooth surface and entire margins on Sabouraud dextrose agar. On CHROMagar Candida, it initially produces pink colonies, which later transform into dark metallic blue, resembling those of *C. tropicalis* [11]. Unlike *C. albicans*, most MALDI-TOF MS systems have difficulties identifying *C. blankii.* VITEK 2 misidentified the yeast as *Candida ciferrii* and *Trichosporon asahii* for Chowdhary et al. [8] and as *Stephanoascus ciferrii* for Al-Haqqan et al. [9]. This may suggest that the spectra of this organism are not present in the libraries of either system. Adding the spectra could enhance the ability to identify the organisms and improve turnaround time. Identification of *C. blankii* often required sending the isolate to a reference laboratory for further investigation. This was the case here, where identification was ultimately achieved through molecular sequence analyses, as ITS sequencing is not available in-house in our lab; this resulted in delaying diagnosis for front-line clinicians.

In turn, therapy for *C. blankii* infections is often initiated empirically. As evidenced by our findings, *C. blankii* exhibits a higher MIC to azoles and lower MIC to echinocandins and polyenes. This was consistent with other cases of *C. blankii* infection in the literature. Still, a dearth of data about the optimal treatment for patients with *C. blankii* infection remains, and no breakpoints for *C. blankii* have yet been established (as is typical for rare yeasts). The use of amphotericin, voriconazole, or itraconazole appears to have favorable outcome based on the reported cases.

## 5. Conclusion

The cases we present in two adults highlight the pathogenic potential for *C. blankii* in humans. In prior reports, as in ours, challenges presented with the emergence of new fungal pathogens are that of an accurate and timely identification of the organism using resources available to most laboratory settings, and in determining its susceptibility patterns to common antifungal agents. Further study into *C. blankii* infections in humans is needed. This would create an opportunity to improve existing laboratory-based assays and would aid clinicians in the more expedient institution of appropriate therapeutic management for patients.

## Figures and Tables

**Table 1:** Reported cases of *C. blankii* isolated in hospitalized patients.

Age	Nation	Co-morbidities	Site	Drug susceptibilities	Treatment	Outcome	Misidentification
2-3 days [8]	India	VLBW, IUGR, sepsis, mechanical ventilation, thrombocytopenia	Blood	FLC 8 μg/mL CAS 1 μg/mL AMB 0.5 μg/mL	FLC × 10 days	Deceased	∗
2-3 days [8]	India	LBW, IUGR, sepsis, thrombocytopenia	Blood	FLC 8 μg/mL CAS 1 μg/mL AMB 0.25 μg/mL	FLC × 14 days	Survived	∗
2-3 days [8]	India	Preterm, LBW, sepsis, thrombocytopenia	Blood	FLC 8 μg/mL CAS 1 μg/mL AMB 0.25 μg/mL	FLC × 14 days	Survived	∗
2-3 days [8]	India	Preterm, LBW, IUGR, thrombocytopenia, maternal preeclampsia, antepartum hemorrhage	Blood	FLC 8 μg/mL CAS 1 μg/mL AMB 0.25 μg/mL	FLC × 12 days	Survived	∗
2-3 days [8]	India	VLBW, persistent hypoglycemia, sepsis, mechanical ventilation	Blood	FLC 8 μg/mL CAS 1 μg/mL AMB 0.25 μg/mL	FLC × 6 days	Deceased	∗
2-3 days [8]	India	Preterm, ELBW, sepsis, mechanical ventilation	Blood	FLC 8 μg/mL CAS 2 μg/mL AMB 0.25 μg/mL	FLC × 10 days	Deceased	∗
2-3 days [8]	India	Preterm, VLBW, sepsis, persistent hypoglycemia, mechanical ventilation	Blood	FLC 8 μg/mL CAS 2 μg/mL AMB 0.25 μg/mL	FLC × 10 days	Survived	∗
2-3 days [8]	India	Persistent hypoglycemia, thrombocytopenia, mechanical ventilation	Blood	FLC 8 μg/mL CAS 1 μg/mL AMB 0.25 μg/mL	FLC × 5 days	Deceased	∗
2-3 days [8]	India	Preterm, ELBW, thrombocytopenia, sepsis, mechanical ventilation	Blood	FLC 8 μg/mL CAS 2 μg/mL AMB 0.25 μg/mL	FLC × 21 days	Survived	∗
27 weeks [9]	Kuwait	Preterm, necrotizing enterocolitis	Blood	FLC 12–16 μg/mL CAS 0.25–0.5 μg/mL AMB 0.125 μg/mL	AMB and CAS	Deceased	Stephanoascus ciferrii
14 years [10]	Argentina	Cystic fibrosis	Respiratory (BAL)	All tested < 0.13 μg/mL	ITC	Survived	Isolate could not be identified by Vitek2
16 years [11]	Brazil	Cystic fibrosis post lung transplant. Pulmonary aspergillosis	Blood	FLC 16 μg/mL ANF 1 μg/mL AMB 0.5 μg/mL	MFG × 14 days	Survived	Isolate could not be identified by MALDI-TOF MS
63 years [12]	USA	Endocarditis, perinephric abscess, hypertension, dyslipidemia, diabetes mellitus, GERD, CVA, chronic lower back pain, pulmonary aspergillosis	Blood	FLC 16 μg/mL CAS 1 μg/mL AMB 0.5 μg/mL	AMB × 4 weeks and MFG × 12 weeks followed by VRC suppression × 9 mth	Survived	Routine lab methods were inconclusive
76 years [13]	USA	COVID-19 pneumonia, hypertension, diabetes mellitus, coronary artery disease, peripheral arterial disease, heart failure	Blood	FLC 256 μg/mL CAS 0.25 μg/mL AMB 0.064 μg/mL	VRC	Deceased	Trichosporon inkin, Malassezia furfur, and Empedobacter brevis
70 years [14]	France	Secondary prophylaxis, pulmonary aspergillosis	Bone	FLC 8 μg/mL CAS 0.06 μg/mL AMB 0.5 μg/mL ISZ 0.25 μg/mL MFG 0.125 μg/mL POS 0.5 μg/mL VRC 0.5 μg/mL 5-FC ≤ 0.125	VRC 10 weeks and F-FC × 2 weeks followed by POS × 2 mth then CAS × 3 mth	Survived	Stephanoascus ciferrii

*Note:* AMB, amphotericin B; ANF, anidulafungin; CAS, caspofungin; FLC, fluconazole; 5-FC, 5-fluorocytosine; GERD, gastroesophageal reflux disease; ISZ, isavuconazole; ITC, itraconazole; IUGR, intrauterine growth restriction; MFG, micafungin; POS, posaconazole; VRC, voriconazole, ELBW, extremely low birth weight (< 1000 g); LBW, low birth weight (< 2500 g); VLBW, very low birth weight (< 1500 g).

Abbreviation: CVA, cerebrovascular accident.

^∗^In the case presented by Spruijtenburg et al. [7]. 5 isolates were misidentified as *Candida ciferrii* and another 5 as *Trichosporon asahii* across all neonates.
